# Topological indices and QSPR analysis of drug molecules from different therapeutic classes

**DOI:** 10.1038/s41598-026-62242-7

**Published:** 2026-07-24

**Authors:** Zeeshan Saleem Mufti, Umm e Rubab, Aiedh Mrisi Alharthi, Gamachu Adugna Ganati

**Affiliations:** 1 Department of Mathematics and Statistics, The University of Lahore, Lahore, Pakistan; 2 Department of Mathematics, Turabah University College, Taif University, Taif, Saudi Arabia; 3 Department of Mathematics, Wallaga University, Nekemte, Ethiopia

**Keywords:** Neighborhood based topological indices, Chemical graph theory, Drug molecular structures, Quantitative structure–property relationship (QSPR), Regression modeling, Chemistry, Materials science, Mathematics and computing

## Abstract

Typically, Quantitative Structure–Property Relationship (QSPR) models are created for compounds related to a particular therapeutic use and might be restricted in scope. To explore the wider use of neighbourhood connectivity descriptors, ten drug molecules, encompassing a wide range of chemical and therapeutic classes, were chosen: anti-inflammatory, anti-bacterial, anti-viral, anti-hypertensive, anesthetic, anti-depressant and neuromuscular agents. The molecular structures were represented as graphs, with atoms represented by the vertices and chemical bonds represented by the edges, and various neighbourhood degree-based topological indices were calculated. Linear, quadratic and cubic QSPR regression models were used to correlate these descriptors with nine experimentally determined physicochemical properties such as boiling point, density, enthalpy of vaporization, flash point, refractive index, molar refractivity, polarizability, surface tension and molar volume. The coefficient of determination ($\textrm{R}^{\circ }$) was used to evaluate the model performance. The results show that neighbourhood-based descriptors are able to capture molecular structural information and make reliable prediction of properties on structurally diverse drug molecules. The results provide good justification to use these descriptors in general for chemical graph theory and in QSPR studies without any particular disease or therapeutic indication.

## Background and literature review

One of the basic tools of chemical graph theory is the ability to map molecular structures into mathematical structures, where atoms correspond to vertices and chemical bonds correspond to edges^1^. This abstraction makes it possible to use discrete mathematical tools to analyze the architecture of a molecule in a systematic way, providing a way to compare the structure of structurally diverse molecules, while avoiding the expensive experimental measurements of the molecule^2^. These graphs are used to provide quantitative representations of molecular structure, known as topological indices, which can then be correlated with known physicochemical properties of molecules, such as their molecular function or toxicity, to numerically characterize the molecular structure^1^. Neighborhood degree based topological indices have emerged, among the different classes of descriptors, to be of special interest as these show not only the degree of the central atom but also the degrees of its immediate neighbours. This extra structural information helps them distinguish between isomers and to capture subtle steric and electronic effects that are not picked up by simpler indices based on the degree of the molecule^3,4^.

These structural descriptors can be correlated with experimental properties like boiling points, density, molar refractivity or polarizability through the standard statistical tools of the Quantitative Structure Property Relationship (QSPR) framework. By using regression analysis, researchers can make accurate estimation of the molecular properties, do not need to go to the tedious laboratory procedure, and speed up the early stage drug discovery, as well as to decrease the experimental cost^5^. The literature more recently confirms the validity and generality of these descriptors in different therapeutic fields, but in most cases only in single disease settings.

For example, Ravi and Chidambaram^1^ were able to use neighborhood degree-based topological descriptors to model the physicochemical and pharmacological properties of drugs used to treat Parkinson’s disease, a task that showed that the topology of molecules plays an important role in the performance of the drugs. Abubakar et al.^2^ took this idea further by integrating neighborhood degree-based indices with Support Vector Regression (SVR) to predict antituberculosis drugs, demonstrating a remarkable ability to model intricate, non-linear relationships and boosting the predictive power of traditional linear models. Balasubramaniyan et al.^3^ found that neighborhood descriptors based on degree of neighborhood greatly improve structure property relationships in anti asthma agents, and that by using regression and machine learning models, these descriptors can be used for drug optimization and virtual screening.

In addition to the conventional degree counts, Zaman et al. used eccentricity based neighborhood indices for the analysis of QSPR of PAH derived drugs and found it effective in representing structural difference and predicting physicochemical properties of drugs^4^. Altassan et al.^5^ examined whole neighborhood indices used in breast cancer therapeutics where they found that there were strong correlations with physicochemical properties using standard regression models. But, Raza et al.^6^ observed that conventional descriptors were not very effective when used for complex polycyclic structures, so they designed the specialized Neighborhood Face Index, which would be more effective to predict the physical properties of a polycyclic chemical compound.

Recently, these tools have been expanded to infectious diseases. Kara et al.^7^ used the topological indices derived from neighborhood eccentricity for the classification of COVID-19 drugs and found that there is a good correlation between the topological indices and the boiling point and molecular volume of the drugs, and importantly, they used Leave One Out Cross Validation (LOOCV) for the validation of their models. This very strict validation method, however, is not widely used in the general literature. Das et al.^8^ carried out QSPR modelling of Molnupiravir and other antiviral drugs for treating COVID-19 patients and concluded that Topological Indices are an effective tool to predict the physicochemical properties of the compounds. Similar descriptor-based methods were used by Tamilarasi and Balamurugan^9^ to compare the pharmacokinetic and toxicity properties of antifungal drugs using topological descriptors. Kirmani and Ali^10^ employed topological descriptors in the development of a QSPR model for the dextran-quercetin conjugates used in cancer treatment, whereas Arockiaraj et al.^11^ applied degree and neighborhood degree sum-based descriptors in the skin cancer drug structures, and showed the superiority of neighborhood descriptors over the classical descriptors.

Notably, these indices are not only useful in the pharmaceutical industry. It is not only applicable to drug design and these descriptors have been also used in other areas, such as predicting corrosion inhibition efficiency of organic compounds (SVR, GBR, KNN) as investigated by Akrom et al.^12^. Rahul and Clement^13^ used molecular descriptors and information entropies to conduct QSPR analysis on carbon allotropes. Neighborhood degree-based descriptors and entropy measures were also applied to energetic properties prediction of the family of the triphenylenoids, as well as to melamine-based structures by Augustine and Santiago^14^ and Jyothish et al.^15^ respectively, showing the versatility of these descriptors in chemically diverse molecular systems.

### The critical gap and necessity of the present study

However, one glaring disadvantage remains: this large and varied oeuvre is entirely uncoordinated. Nearly all prior investigations whether focused on Parkinson’s disease^1^, tuberculosis^2^, asthma^3^, PAHs^4^, breast cancer^5^, polycyclic compounds^6^, COVID-19^7,8^, antifungals^9^, cancer conjugates^10^, skin cancer^11^, or non-pharmaceutical domains ^12–15^—are anchored to a single disease indication or a narrowly defined therapeutic class. Therefore, the usefulness of a set of indices for prediction is guaranteed only under that particular clinical or chemical circumstance. These descriptors are so far not well tested if they actually explain and predict the effect of a structurally and pathologically mixed set of drugs^7,11^.

This implicit constraint is what we call “disease specific bias”, a limitation that decreases the perceived applicability of topological indices, and discourages their use in a broad-spectrum of computational screening workflows. Moreover, though a few recent works have used more sophisticated regression models and test procedures^2,7^, most of the available models are still based on simple linear regressions without a thorough internal testing procedure. The systematic use of LOOCV for model stability evaluation in very diverse data sets is still barely mentioned in the literature^7^. Furthermore, because of the lack of transparency in the computational implementation (as these pipelines are not open-source and not reproducible), the other research groups cannot easily benefit from the use and validation of these models.

The present study intentionally uses a heterogeneous set of data to directly address these interrelated constraints. Ten agents were selected for screening all not pharmacologically related from the fields of veterinary anti-inflammatory therapy (tepoxalin), diagnostic microbiology (nitrocefin), bone metabolism (neridronic acid), anesthesiology (tiletamine), neurology and psychiatry (filorexant, rapastinel), infectious diseases (radezolid, ingavirin), muscle activation (tirasemtiv), and cardiovascular therapy (urapidil). This intentional selection is used as a test bed to investigate the generalizability of descriptors to several pathological domains. All drugs, including specific clinical indications and physicochemical properties are listed in the Materials and Methds.

We recognize that our sample size (n=10) is small, but this is a proof of concept study to validate whether neighborhood connectivity descriptors retain predictive power in the very broad chemical space.The structural and therapeutic diversity of these compounds imposes a far more stringent test of descriptor universality than would be possible using a large but structurally homogeneous dataset. Molecular structures were obtained from the ChemSpider database^16^ and all the neighborhood degree based topological indices were calculated from the structures using a custom made Python script (Python version 3.10) developed with the help of the NetworkX library^17^. The QSPR regression analyses (linear, quadratic and cubic) were done using the IBM SPSS Statistics package^18^. Model stability was thoroughly tested via LOOCV (using Python). This work aims to show this, by explicitly listing the type of diseases and by testing our models across a non related set of therapeutic areas, making our neighborhood connectivity descriptors not only a disease-specific tool but also a generic structural quantifier that can be used to aid in the drug discovery process beyond one pathological condition.

## Preliminaries and methodology

### Definition 1

A graph *G=(V,E)* is a triple consisting of a vertex set *V*(*G*), an edge set *E*(*G*), and a relation that associates with each edge two vertices (not necessarily distinct) called its endpoints.

### Definition 2

Let *G=(V,E)* be a simple graph and let *u ∈ V(G)* be a vertex. The degree of a vertex *u*, denoted by $d_G(u)$, is the number of edges incident to *u*.The neighborhood of *u* is denoted by *N*(*u*) and consists of all vertices adjacent to *u*.The *neighborhood degree* of a vertex *u*, denoted by $\delta _G(u)$, is defined as the sum of the degrees of all vertices in the neighborhood of *u*. Mathematically, it is given by

$$\delta _G(u)=\sum _{v \in N(u)} d_G(v).$$

### Definition 3

The neighborhood index is introduced by Kulli in^19^, is defined as:

1 $$\begin{aligned} NM_1(G) = \sum _{uv \in E(G)} \big [\, \delta _G(u)\,+ \delta _G(v) \,\big ] \end{aligned}$$

### Definition 4

The neighborhood ABC index of a graph in^20^ is defined as:

2 $$\begin{aligned} ABC_N(G) = \sum _{uv \in E(G)} \sqrt{\frac{\delta _G(u) + \delta _G(v) - 2}{\delta _G(u)\,\delta _G(v)}} \end{aligned}$$

### Definition 5

The neighborhood second Zagreb index is defined in^21^ as:

3 $$\begin{aligned} NM_2(G) = \sum _{uv \in E(G)} \big [\, \delta _G(u)\delta _G(v) \,\big ] \end{aligned}$$

### Definition 6

The neighborhood forgotten index^22^ is defined as:

4 $$\begin{aligned} NF(G) = \sum _{uv \in E(G)} \big [\, \delta _G(u)^2 + \delta _G(v)^2 \,\big ] \end{aligned}$$

### Definition 7

The first neighborhood degree index ($ND_1$) is expressed as in^23^:

5 $$\begin{aligned} ND_{1}(G) = \sum _{uv \in E(G)} \sqrt{\delta _G(u) \, \delta _G(v)} \end{aligned}$$

### Definition 8

The second neighborhood degree index ($ND_2$) is explained as in^23^

6 $$\begin{aligned} ND_{2}(G) = \sum _{uv \in E(G)} \frac{1}{\sqrt{\delta _G(u) + \delta _G(v)}} \end{aligned}$$

### Definition 9

The third neighborhood degree index ($ND_3$) is described as in^23^

7 $$\begin{aligned} ND_{3}(G) = \sum _{uv \in E(G)} \delta _G(u)\,\delta _G(v)\,[\delta _G(u) + \delta _G(v)] \end{aligned}$$

### Definition 10

The fourth neighborhood degree index ($ND_4$)^23^

8 $$\begin{aligned} ND_{4}(G) = \sum _{uv \in E(G)} \frac{1}{\sqrt{\delta _G(u)\,\delta _G(v)}} \end{aligned}$$

### Definition 11

The ratio based relationship between the neighbourhood degrees of closest vertices in a graph is by Mondal et. al^23^ with the name fifth neighbourhood degree index ($ND_5$) is defined as

9 $$\begin{aligned} ND_{5}(G) = \sum _{uv \in E(G)} \left[ \frac{\delta _G(u)}{\delta _G(v)} + \frac{\delta _G(v)}{\delta _G(u)}\right] . \end{aligned}$$

Ten drug compounds were selected and their molecular structures were retrieved from ChemSpider^16^. The molecular graph was manually drawn from the structural formula for each compound. The neighbourhood degree-based topological indices were calculated with a custom Python script (Python 3.10) that utilises the NetworkX library for graph operations^17^. IBM SPSS Statistics^18^ was used to perform all QSPR regression analyses (linear, quadratic, and cubic). The model stability was tested using Leave-One-Out Cross-Validation (LOOCV) in Python. Topological indices are useful throughout the entire procedure, starting with the development of a new drug, and they show various attributes of pharmaceuticals. In this section, we chose nine neighborhood topological indices and nine physical attributes of ten drugs to determine the composition of Tepoxalin, Nitrocefin, Neridronic acid, Tiletamine, Filorexant, Rapastinel, Radezolid, Tirasemtiv, Urapidil, and Ingavirin. The brief introduction of these disease has been mentioned above. This section also explains how to determine the values for each of the ten properties listed above and shows the chemical structures of all ten.Drug molecules that were used in this research were chosen on the basis of the access to reliable data on the physicochemical properties and the diversity of their structure. These compounds are various pharmaceutical agents whose molecular structures and complexities might differ hence suitable in assessing the usefulness of the degree of neighborhood based topological indices in the QSPR set-up. It is due to the structural differences between these molecules that a valuable study can be made into the question of the relationship between molecular topology and key physicochemical characteristics like boiling point, molar refractivity, polarizability, and molar volume.

### Selected drug molecules

This section provides a summary of selected drug molecules that are of interest in this study. These drugs are pharmaceutical compounds belonging to various classes and are commonly used in the treatment of different diseases. Some of these drugs are already known and approved, while others are in preclinical or investigational stages; however, their physicochemical properties and structural characteristics require further analysis. Computational and mathematical methods assist researchers in gaining a better understanding of their behavior and efficacy. Both approved and investigational drugs are included in this study and are analyzed using topological indices and the QSPR approach. These drugs have been selected to investigate the correlation between molecular structure and physicochemical properties, which aids in drug design and predictive modeling. Here are some brief description of the drugs.

1. Tepoxalin (commonly known as Zubrin) is a non-steroidal anti-inflammatory drug (NSAID) used in veterinary medicine to treat osteoarthritis in dogs by reducing pain and inflammation.
2. Nitrocefin is a chromogenic cephalosporin drug used as a substrate to detect the presence of beta-lactamase enzymes produced by various microorganisms.
3. Neridronic acid (marketed as Nerixia) is an aminobisphosphonate drug that inhibits osteoclastic bone resorption and is used to treat metabolic bone disorders such as osteogenesis imperfecta, Paget’s disease, and complex regional pain syndrome (CRPS).
4. Tiletamine is a dissociative anesthetic drug used in veterinary medicine for anesthesia and immobilization, commonly administered in combination with zolazepam (as Telazol) to reduce muscle spasms.
5. Filorexant (*MK-6096*) is an investigational drug that acts as a dual orexin receptor antagonist (DORA), targeting OX$_1$ and OX$_2$ receptors, and is being developed for the treatment of insomnia, depression, migraines, and diabetic neuropathy.
6. Rapastinel (formerly known as GLYX-13) is an investigational drug and a fast-acting intravenous antidepressant that functions as an NMDA receptor modulator, designed to produce rapid therapeutic effects without psychotomimetic side effects.
7. Radezolid is an antibacterial drug that has been investigated for the treatment of abscesses, bacterial skin infections, streptococcal infections, and staphylococcal skin infections.
8. Tirasemtiv (CK-2017357) is an investigational drug that acts as a fast skeletal muscle troponin activator, enhancing calcium sensitivity in skeletal muscles.
9. Urapidil is an antihypertensive drug, a uracil derivative substituted with phenylpiperazine, primarily used in the treatment of severe hypertension and hypertensive emergencies.
10. Ingavirin is an antiviral drug developed in Russia, used for the treatment and prevention of influenza A and B, COVID-19, and other acute respiratory viral infections.

**Figure:**
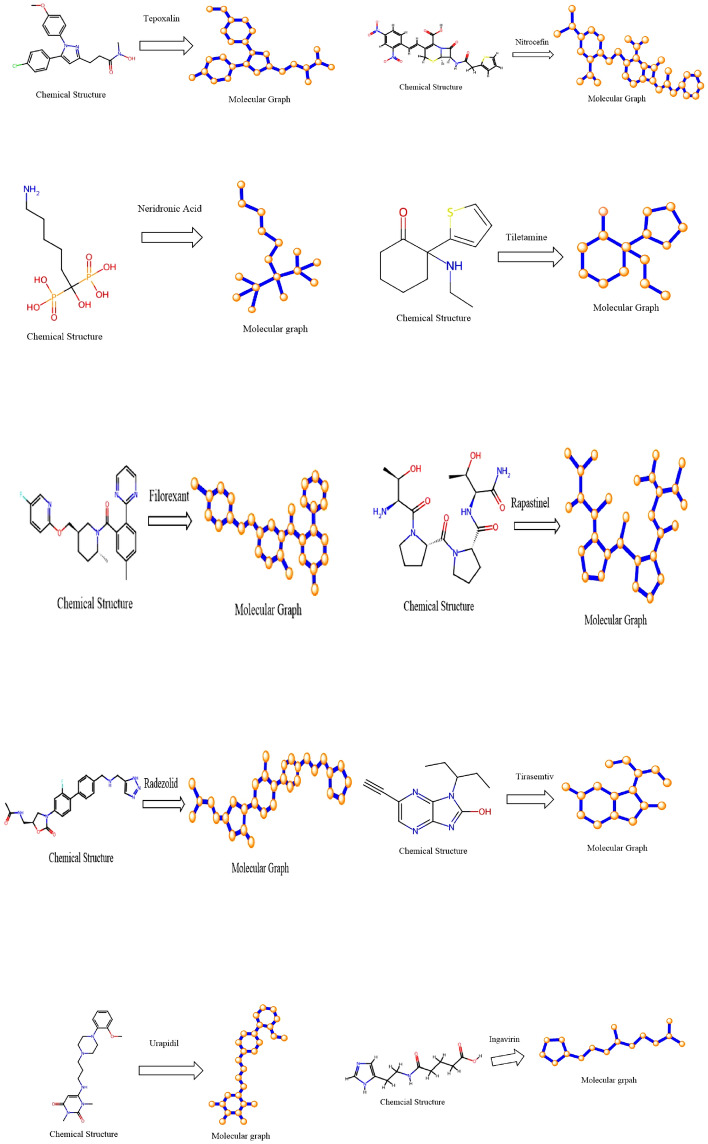


Edge distribution analysis was used to analyze the structural connectivity patterns of ten biochemical compounds in this study. A neighborhood degree-based edge distribution approach was used to systematically capture the local structural variation for each molecular graph. This approach allows for a uniform encoding of edge partitioning based on the degree combinations of the edges’ vertices, so that all drug networks examined can be analyzed comparatively. For clarity and easy comparison, all the edge distributions calculated are compiled in a single comprehensive table. The total neighborhood degree-based edge distributions for all ten biochemical compounds, Tepoxalin, Nitrocefin, Neridronic acid, Tiletamine, Filorexant, Rapastinel, Radezolid, Tirasemtiv, Urapidil and Ingavirin, are presented in Table 1. The rows of the table represent individual compounds, and the edge partitions and their frequencies are presented in a structured format within the same row. This unified picture helps to compare the structural connectivity patterns between different molecular graphs and also shows the different structural connectivity configurations of the neighborhoods of the selected biochemical compounds in Table 1.

**Table 1:** Edge partition of all drugs by neighbourhood degree.

**Drug**	**Edge Partitions and Frequencies**
Tepoxalin	$$\begin{array}{|c|c|c|c|c|c|c|c|c|c|c|} \hline (2\,\, 4) & (3\,\, 5) & (3\,\, 6) & (4\,\, 6) & (5\,\, 5) & (5\,\, 6) & (5\,\, 7) & (6\,\, 6) & (6\,\, 8) & (7\,\, 8) & (8\,\, 8)\\ \hline 1 & 3 & 1 & 1 & 7 & 5 & 4 & 2 & 2 & 2 & 1\\ \hline \end{array}$$
Nitrocefin	$$\begin{array}{|c|c|c|c|c|c|c|c|c|c|c|c|c|c|c|c|c|} \hline (3\,\, 5) & (5\,\, 7) & (5\,\, 5) & (6\,\, 7) & (6\,\, 8) & (7\,\, 8) & (5\,\, 8) & (7\,\, 9) & (5\,\, 9) & (9\,\, 9) & (8\,\, 9) & (8\,\, 8) & (3\,\, 7) & (5\,\, 6) & (6\,\, 6) & (4\,\, 5) & (4\,\, 4)\\ \hline 7 & 6 & 3 & 1 & 2 & 2 & 2 & 2 & 1 & 1 & 1 & 1 & 1 & 4 & 1 & 2 & 1\\ \hline \end{array}$$
Neridronic Acid	$$\begin{array}{|c|c|c|c|c|c|c|c|} \hline (2\,\, 3) & (3\,\, 4) & (4\,\, 4) & (4\,\, 6) & (6\,\, 11) & (7\,\, 11) & (11\,\, 4) & (7\,\, 4)\\ \hline 1 & 1 & 2 & 1 & 1 & 2 & 1 & 6\\ \hline \end{array}$$
Tiletamine	$$\begin{array}{|c|c|c|c|c|c|c|c|c|c|c|} \hline (2\,\, 3) & (3\,\, 6) & (3\,\, 7) & (4\,\, 4) & (4\,\, 5) & (4\,\, 6) & (5\,\, 7) & (5\,\, 8) & (6\,\, 10) & (7\,\, 10) & (8\,\, 10)\\ \hline 1 & 1 & 1 & 2 & 3 & 1 & 1 & 2 & 2 & 1 & 1\\ \hline \end{array}$$
Filorexant	$$\begin{array}{|c|c|c|c|c|c|c|c|c|c|c|c|c|} \hline (3\,\, 5) & (3\,\, 6) & (3\,\, 7) & (4\,\, 4) & (4\,\, 5) & (5\,\, 5) & (5\,\, 6) & (5\,\, 7) & (5\,\, 8) & (6\,\, 6) & (6\,\, 8) & (7\,\, 8) & (8\,\, 8)\\ \hline 2 & 1 & 1 & 2 & 2 & 8 & 7 & 2 & 1 & 1 & 3 & 3 & 1\\ \hline \end{array}$$
Rapastinel	$$\begin{array}{|c|c|c|c|c|c|c|c|c|c|c|c|} \hline (3\,\, 5) & (5\,\, 7) & (7\,\, 3) & (7\,\, 7) & (7\,\, 8) & (5\,\, 8) & (5\,\, 4) & (8\,\, 8) & (7\,\, 3) & (6\,\, 8) & (6\,\, 3) & (6\,\, 6)\\ \hline 6 & 1 & 2 & 1 & 3 & 6 & 4 & 2 & 1 & 2 & 1 & 1\\ \hline \end{array}$$
Radezolid	$$\begin{array}{|c|c|c|c|c|c|c|c|c|c|c|c|} \hline (3\,\, 4) & (3\,\, 6) & (4\,\, 4) & (4\,\, 5) & (5\,\, 5) & (5\,\, 6) & (5\,\, 7) & (5\,\, 8) & (6\,\, 6) & (6\,\, 7) & (6\,\, 8) & (7\,\, 8)\\ \hline 2 & 2 & 1 & 5 & 4 & 7 & 3 & 1 & 4 & 1 & 3 & 2\\ \hline \end{array}$$
Tirasemtiv	$$\begin{array}{|c|c|c|c|c|c|c|c|c|c|c|} \hline (3\,\, 5) & (4\,\, 2) & (5\,\, 5) & (6\,\, 8) & (5\,\, 6) & (5\,\, 7) & (7\,\, 8) & (7\,\, 6) & (6\,\, 6) & (7\,\, 4) & (6\,\, 3)\\ \hline 1 & 2 & 2 & 2 & 1 & 1 & 1 & 2 & 2 & 2 & 1\\ \hline \end{array}$$
Urapidil	$$\begin{array}{|c|c|c|c|c|c|c|c|c|c|c|c|c|c|} \hline (2\,\, 4) & (4\,\, 7) & (5\,\, 7) & (7\,\, 8) & (4\,\, 5) & (4\,\, 4) & (5\,\, 8) & (5\,\, 5) & (5\,\, 6) & (6\,\, 7) & (7\,\, 7) & (3\,\, 7) & (6\,\, 6) & (3\,\, 6)\\ \hline 1 & 1 & 4 & 2 & 4 & 2 & 1 & 2 & 3 & 2 & 3 & 3 & 1 & 1\\ \hline \end{array}$$
Ingavirin	$$\begin{array}{|c|c|c|c|c|c|c|} \hline (4\,\, 3) & (4\,\, 4) & (4\,\, 5) & (5\,\, 3) & (5\,\, 5) & (5\,\, 6)\\ \hline 2 & 1 & 7 & 1 & 2 & 3\\ \hline \end{array}$$

### Chemical configuration and physical behaviour of drugs

Chemical compounds have different properties so dissuession is only on ten properties in Table 2: Boiling point(BP), Density(D), Enthalpy of Vaporization(EV), Flash Point(FP), Index of Refraction(IR), Molar Refractivity(MR), Polarizability(P), Surface Tension(ST), and Molar Volume(MV). In following Table 3 neighbourhood topological indices like Neighborhood Zagreb Index 1 (MN1), Neighborhood ABC Index (NABC), Neighbourhood second Zagreb index (NM2), Forgotten topological index (FN1). The first index $\mathcal{N}\mathcal{D}_1$, the second index $\mathcal{N}\mathcal{D}_2$, the third index $\mathcal{N}\mathcal{D}_3$, the fourth index $\mathcal{N}\mathcal{D}_4$, and the fifth index $\mathcal{N}\mathcal{D}_5$ of each drug are presented in separate rows.

**Table 2:** Parameters of drugs.

Sr no	Drugs name	BP	D	EV	FP	IR	MR	P	ST	MV
1	Tepoxalin	573	1.3	90.3	300.3	1.61	105.4	41.8	46.8	303.8
2	Nitrocefin	872	1.7	132.8	481.2	1.749	125.3	49.7	95.3	307.7
3	Neridronic acid	600.1	1.7	102.5	316.7	1.569	54.8	21.7	95.8	167.1
4	Tiletamine	352.6	1.1	59.7	167.1	1.556	63.7	25.2	43.8	198
5	Filorexant	540.2	1.2	81.3	280.5	1.576	115.6	45.8	48.2	349.4
6	Rapastinel	844.2	1.4	139.5	464.4	1.59	101.8	40.3	70.7	301.7
7	Radezolid	712.5	1.3	104.2	384.7	1.612	114	45.2	59.2	328.1
8	Tirasemtiv	422.3	1.2	70.3	209.2	1.62	66.2	26.2	43.8	188.4
9	Urapidil	549	1.3	82.9	285.8	1.622	108.3	42.9	59	307.4
10	Ingavirin	649.2	1.3	100.6	346.4	1.554	56.7	22.5	59.5	176.9

**Table 3:** Neighbourhood based topological indices of drugs.

Sr no	Drugs name	$NM_1$	NABC	$NM_2$	FN	N$D_1$	N$D_2$	N$D_3$	N$D_4$	N$D_5$
1	Tepoxalin	320	16.1274	904	1868	158.4625	8.8726	10802	5.5712	60.7929
2	Nitrocefin	444	20.8141	1341	2810	219.064	11.3652	17522	7.0154	80.7202
3	Neridronic acid	172	8.4884	506	1178	82.8688	4.6297	7012	3.0367	34.2532
4	Tiletamine	182	8.9844	546	1192	88.9581	4.9417	7448	3.2119	35.0214
5	Filorexant	378	18.8069	1073	2220	187.2473	10.3406	12890	6.4173	70.8857
6	Rapastinel	344	16.7201	1003	2160	168.2866	9.0545	12802	5.7008	66.2702
7	Radezolid	384	19.417	1073	2214	190.4195	10.7119	12580	6.6813	72.5274
8	Tirasemtiv	187	9.5772	528	1111	92.05	5.2538	6384	3.3948	36.7893
9	Urapidil	332	16.7763	928	1962	163.3475	9.1628	11076	5.7804	64.6726
10	Ingavirin	146	9.4516	335	686	72.5378	5.3334	3166	3.5985	32.8833

The outcomes of the linear, quadratic, and cubic regression models are summarised in a single combined Table 4 for easy comparison. To maintain consistency with the original presentation, all text and numeric values are shown in blue, and the Best Predictor cells are highlighted in bold. For readers who prefer a model-by-model view, the full regression tables are available in the supplementary material.

**Table 4:** Combined outcomes of linear, quadratic, and cubic regression models.

Property	Predictor	Linear		Quadratic		Cubic	
		$R^{2}$	Best Predictor	$R^{2}$	Best Predictor	$R^{2}$	Best Predictor
BP	$NM_{1}$	0.359	–	0.362	–	0.362	–
	NABC	0.368		0.369		0.369	
	$NM_2$	0.353		0.373		0.379	
	FN1	0.366		0.392		0.399	
	$\mathcal{N}\mathcal{D}_1$	0.354		0.357		0.357	
	$\mathcal{N}\mathcal{D}_2$	0.363		0.365		0.365	
	$\mathcal{N}\mathcal{D}_3$	0.353		0.412		0.427	
	$\mathcal{N}\mathcal{D}_4$	0.374		0.378		0.378	
	$\mathcal{N}\mathcal{D}_5$	0.381		0.381		0.382	
FP	$NM_{1}$	0.359	–	0.362	–	0.445	–
	NABC	0.368		0.369		0.428	
	$NM_2$	0.353		0.373		0.470	
	FN1	0.366		0.392		0.491	
	$\mathcal{N}\mathcal{D}_1$	0.354		0.357		0.438	
	$\mathcal{N}\mathcal{D}_2$	0.363		0.365		0.420	
	$\mathcal{N}\mathcal{D}_3$	0.353		0.412		0.521	
	$\mathcal{N}\mathcal{D}_4$	0.374		0.378		0.427	
	$\mathcal{N}\mathcal{D}_5$	0.381		0.382		0.456	
IR	$NM_{1}$	0.432	–	0.481	–	0.481	–
	NABC	0.372		0.431		0.431	
	$NM_2$	0.479		0.512		0.512	
	FN1	0.477		0.506		0.506	
	$\mathcal{N}\mathcal{D}_1$	0.430		0.480		0.480	
	$\mathcal{N}\mathcal{D}_2$	0.365		0.422		0.422	
	$\mathcal{N}\mathcal{D}_3$	0.518		0.529		0.529	
	$\mathcal{N}\mathcal{D}_4$	0.354		0.411		0.411	
	$\mathcal{N}\mathcal{D}_5$	0.397		0.457		0.457	
MR	$NM_{1}$	0.975	NABC	0.983	$ND_1$	0.983	$ND_1$
	NABC	0.980		0.985		0.985	
	$NM_2$	0.941		0.955		0.956	
	FN1	0.913		0.928		0.929	
	$\mathcal{N}\mathcal{D}_1$	0.978		0.986		0.986	
	$\mathcal{N}\mathcal{D}_2$	0.977		0.983		0.983	
	$\mathcal{N}\mathcal{D}_3$	0.853		0.879		0.882	
	$\mathcal{N}\mathcal{D}_4$	0.971		0.977		0.977	
	$\mathcal{N}\mathcal{D}_5$	0.979		0.984		0.984	
P	$NM_{1}$	0.975	NABC	0.983	NABC,$ND_1$	0.983	$ND_1$
	NABC	0.980		0.985		0.985	
	$NM_2$	0.941		0.955		0.955	
	FN1	0.913		0.928		0.929	
	$\mathcal{N}\mathcal{D}_1$	0.978		0.985		0.986	
	$\mathcal{N}\mathcal{D}_2$	0.977		0.983		0.983	
	$\mathcal{N}\mathcal{D}_3$	0.853		0.878		0.882	
	$\mathcal{N}\mathcal{D}_4$	0.971		0.977		0.977	
	$\mathcal{N}\mathcal{D}_5$	0.979		0.983		0.984	
MV	$NM_{1}$	0.886	NABC,$ND_2$	0.892	NABC	0.892	NABC
	NABC	0.918		0.920		0.920	
	$NM_2$	0.830		0.841		0.843	
	FN1	0.8		0.813		0.815	
	$\mathcal{N}\mathcal{D}_1$	0.890		0.895		0.896	
	$\mathcal{N}\mathcal{D}_2$	0.918		0.918		0.919	
	$\mathcal{N}\mathcal{D}_3$	0.719		0.740		0.742	
	$\mathcal{N}\mathcal{D}_4$	0.915		0.916		0.916	
	$\mathcal{N}\mathcal{D}_5$	0.907		0.911		0.913	

## Statistical modeling techniques

To identify which model determines the most suitable relationship between the topological parameters and physicochemical attributes, three regression models were used to explore the relationship.

10 $$\begin{aligned} P = a + b(T) \end{aligned}$$

11 $$\begin{aligned} P = a + b(T) + c(T)^2 \end{aligned}$$

12 $$\begin{aligned} P = a + b(T) + c(T)^2 + d(T)^3 \end{aligned}$$

Where P indicates the physical property,T indicates the topological index, and m,n, and p are constants. Linear and quadratic regression analyses were carried out at two different points using in Table properties and where the proposed indices are shown in Table . In the linear regression model, ’a’ represents a constant,’b’ represents the regression coefficient, F is the F-statistic, R represents the degree of correlation,p shows the P-value, $R^2$ denotes the coefficient of determination, and S represents the standardized residuals. In the quadratic regression model,’f’ represents a constant, ’g’ represents the topological index, and ’h’ is the square of the topological index.

A cubic regression model, where ’i’ is a constant and ’j’ is the regression coefficient derived through the standardization of residuals relative to their standard deviation ’k’ is the square of the topological index, and ’l’ is the cube of the topological index, is used to verify model accuracy and determine the presence of outliers. N signifies the dataset used in the regression analyses. If the P-value is 0.05, then notable statistical behavior is found between the input variables and the outcome variable. If the F-value >2.5, the model represents better outcomes than applying the method without considering the predictor variables. In the analysis of variable relationships, R is close to +1, and the association is positively directed; if -1, then the association is negatively directed values that are above 0.8 usually represent a strong relationship.

**Table 5:** Best predictors and their statistical parameters for linear, quadratic, and cubic regression models.

Model Type	Property	Best Index	Equation	$R^{2}$	*p*-value	RMSE
Linear	MR	NABC	$MR = -1.429 + 0.175\,(\text {NABC})$	0.980	0.001	0.730
	P	NABC	$P = -1.412 + 0.441\,(\text {NABC})$	0.980	0.001	0.724
	MV	NABC	$MV = -2.712 + 0.066\,(\text {NABC})$	0.918	0.001	1.471
		$ND_2$	$MV = -1.410 + 0.036\,(ND_2)$	0.918	0.001	0.804
Quadratic	MR	$ND_1$	$MR = 56.586 + 0.013\,(ND_1)^2 - 0.136\,(ND_1)$	0.986	0.001	7.206
	P	NABC	$P = 4.915 + 0.006\,(\text {NABC})^2 + 0.040\,(\text {NABC})$	0.985	0.001	0.668
		$ND_1$	$P = 55.125 + 0.080\,(ND_1)^2 - 0.696\,(ND_1)$	0.985	0.001	7.259
	MV	NABC	$MV = -7.292 - 0.00007769\,(\text {NABC})^2 + 0.105\,(\text {NABC})$	0.920	0.001	1.559
Cubic	MR	$ND_1$	$MR = 48.155 + 0.00001438\,(ND_1)^3 + 0.009\,(ND_1)$	0.986	0.001	7.193
	MV	NABC	$MV = -6.290 - 0.0000001192\,(\text {NABC})^3 + 0.089\,(\text {NABC})$	0.920	0.001	1.552

**Figure 1:**
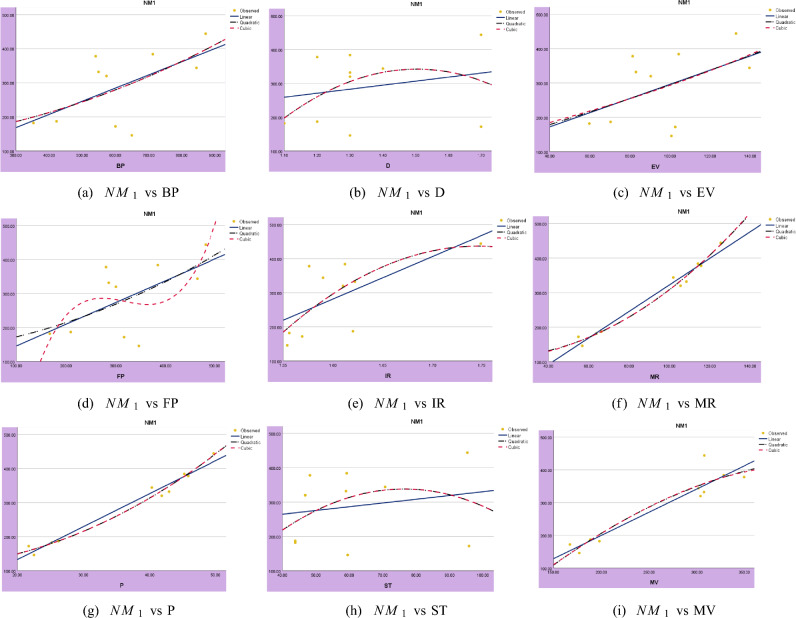
Regression plots corresponding to neighborhood Zagreb index 1.

A compact overview of the best predictors for all three regression models (linear, quadratic, cubic) is given in Table 5. In the linear model, the indices **NABC** and $ND_2$ emerged as the most powerful predictors for molar refractivity, polarizability, and molar volume. Quadratic and cubic models further improved the fit for selected properties, with $ND_1$ and NABC also showing strong performance. A graphical representation of the regression modeling of the neighborhood Zagreb Index ($NM_1$) with linear, quadratic, and cubic regression is shown in Fig. 1. These graphs reveal the closeness of predicted outcomes, and each model describes the relationship between $NM_1$ and the drug properties. The analysis of these regressions shows which model provides the best fit, indicating the stability of the structural properties. Index(NM1) with Linear,Quadratic, and Cubic regression is shown in Fig. 1. These graphs reveal the closeness of predicted outcomes, and each model describes the relationship between the neighborhood Zagreb Index(NM1) and the properties of the drug independent variable and the dependent variables. The analysis of these regressions shows which model provides the best t, indicating the stability of the structural properties.

This Table 6 presents the best-fit cubic regression equations for the properties MR, P, and MV across nine different indices (NM$_1$, NABC, NM$_2$, FN$_1$, ND$_1$–ND$_5$). Where a cubic form was not available for a given property–index combination is indicated. The full set of cubic regression equations for all indices is available in the supplementary materia.

**Table 6:** Best cubic equations for properties MR, P, and MV across all indices.

List of cubic equations for MR, P, and MV	
NM$_1$ – MR: $0.00002766\,(NM_1)^3 + 0.018\,(NM_1)^2 + 99.281$	
NABC – MR: $0.000001107\,(NABC)^3 + 0.001\,(NABC)^2 + 5.206$	
NABC – P: $-0.000002104\,(NABC)^3 + 0.007\,(NABC)^2 + 5.159$	
NABC – MV: $-0.0000001192\,(NABC)^3 + 0.089\,(NABC) - 6.290$	
NM$_2$ – MR: —	
NM$_2$ – P: $0.006\,(NM_2)^3 + 6.621\,(NM_2) + 237.402$	
NM$_2$ – MV: $-0.00001966\,(NM_2)^3 + 8.028\,(NM_2) - 861.124$	
FN$_1$ – MR: $0.001\,(FN_1)^3 + 4.121\,(FN_1) + 616.808$	
FN$_1$ – MV: $-0.00004370\,(FN_1)^3 + 16.959\,(FN_1) - 1772.210$	
ND$_1$ – MV: $-0.000002179\,(ND_1)^3 + 1.135\,(ND_1) - 108.839$	
ND$_2$ – MR: $-0.0000003487\,(ND_2)^3 + 0.001\,(ND_2)^2 + 2.978$	
ND$_2$ – P: $-0.0000007446\,(ND_2)^3 + 0.004\,(ND_2)^2 + 2.951$	
ND$_2$ – MV: $-0.00000004982\,(ND_2)^3 + 0.045\,(ND_2) - 2.906$	
ND$_3$ – MR: $0.008\,(ND_3)^3 - 0.307\,(ND_3) + 5302.365$	
ND$_3$ – P: $0.129\,(ND_3)^3 - 1.905\,(ND_3)^2 + 5296.032$	
ND$_4$ – P: $0.129\,(ND_4)^3 - 1.905\,(ND_4)^2 + 2596.032$	
ND$_4$ – MV: $-0.00000003012\,(ND_4)^3 + 0.027\,(ND_4) - 1.396$	
ND$_5$ – MR: $-0.000006151\,(ND_5)^3 + 0.005\,(ND_5)^2 + 19.186$	
ND$_5$ – MV: $-0.0000007913\,(ND_5)^3 + 0.406\,(ND_5) - 34.032$	

**Figure 2:**
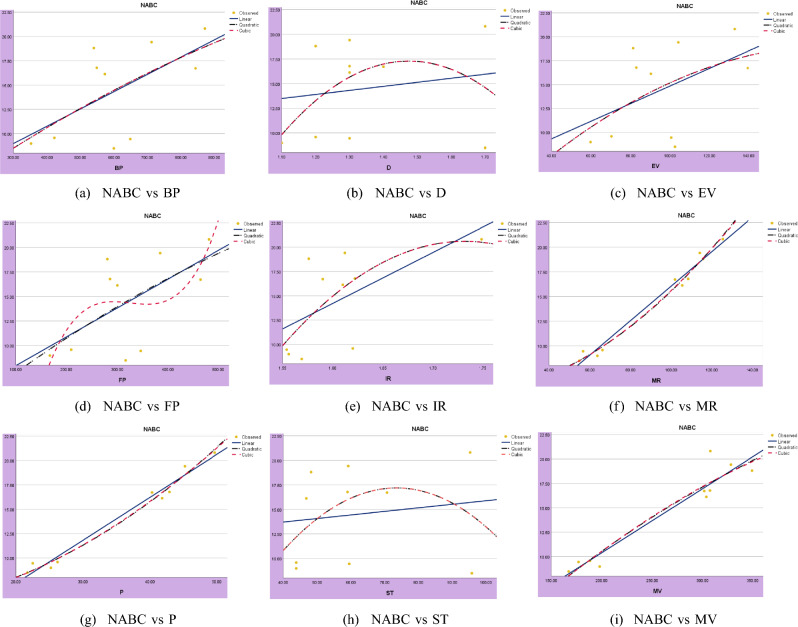
Regression plots corresponding to neighborhood ABC index.

The full set of cubic regression equations for all indices is available in the Supplementary Material. A diagrammatic representation of the regression model of the neighborhood ABC index with Linear, Quadratic, and cubic statistical models.These graphs show how closely each model re ects the relationship between the independent variable and dependent variables. Based on the regression results, we identi ed which model shows a strong relationship and gives a high prediction accuracy of the neighborhood ABC index, as shown in Fig. 2.

**Figure 3:**
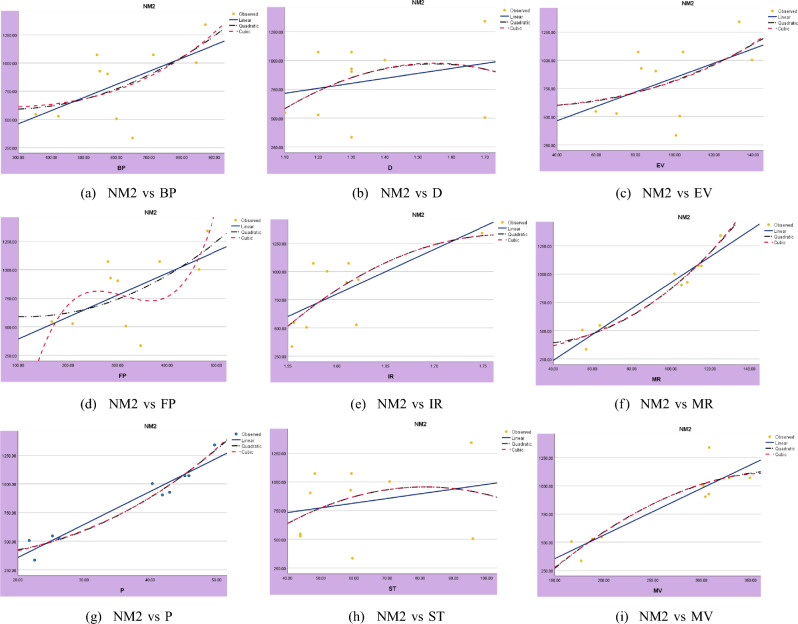
Regression plots corresponding to neighbourhood second Zagreb index.

**Figure 4:**
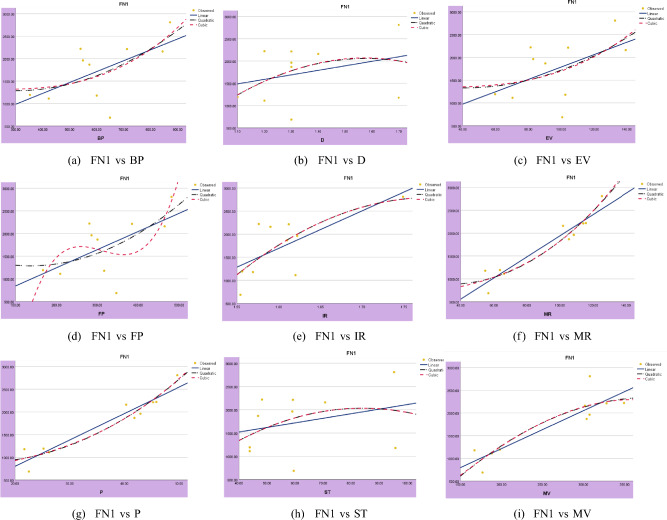
Regression plots corresponding to forgetting neighbourhood index.

**Figure 5:**
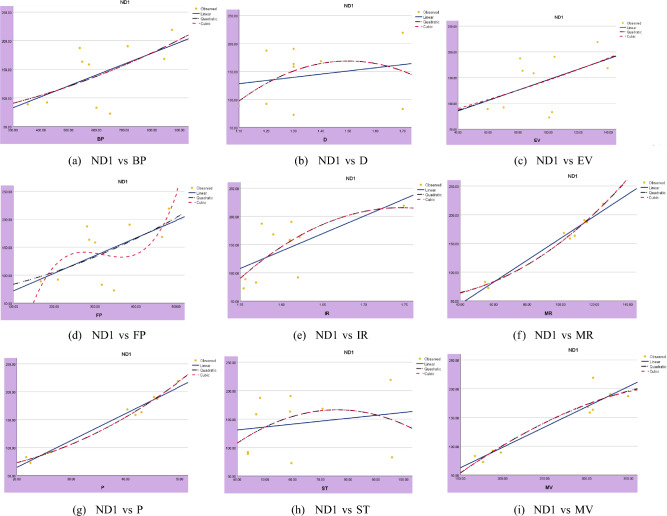
Regression plots corresponding to ND1 index.

**Figure 6:**
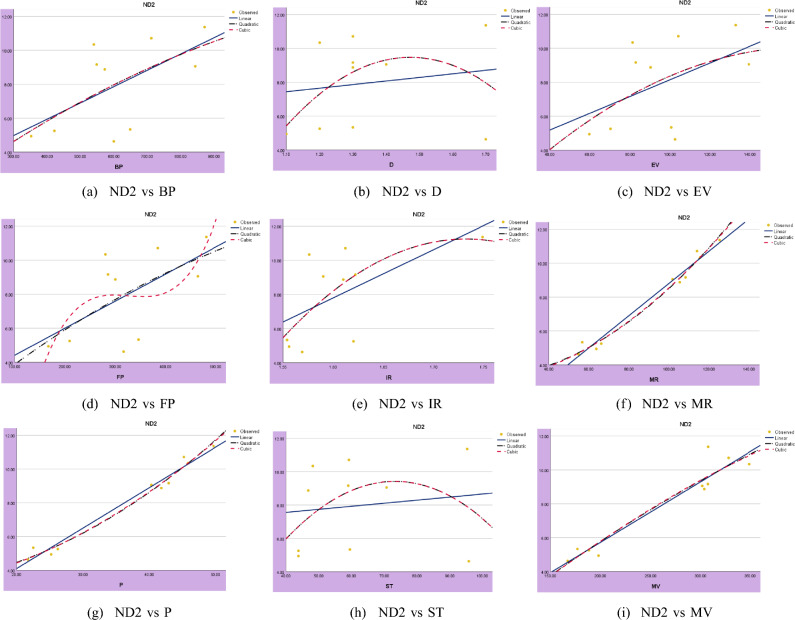
Regression plots corresponding to ND2 index.

**Figure 7:**
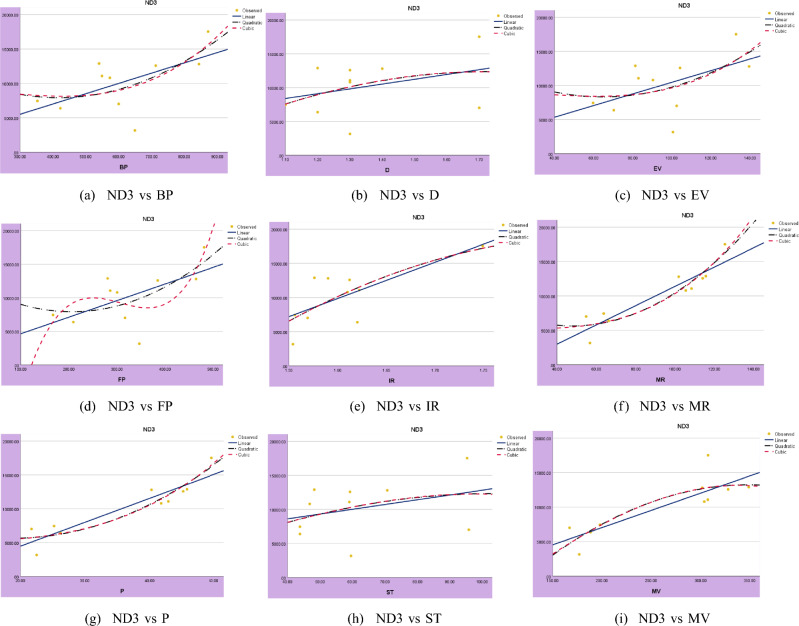
Regression plots corresponding to ND3 index.

**Figure 8:**
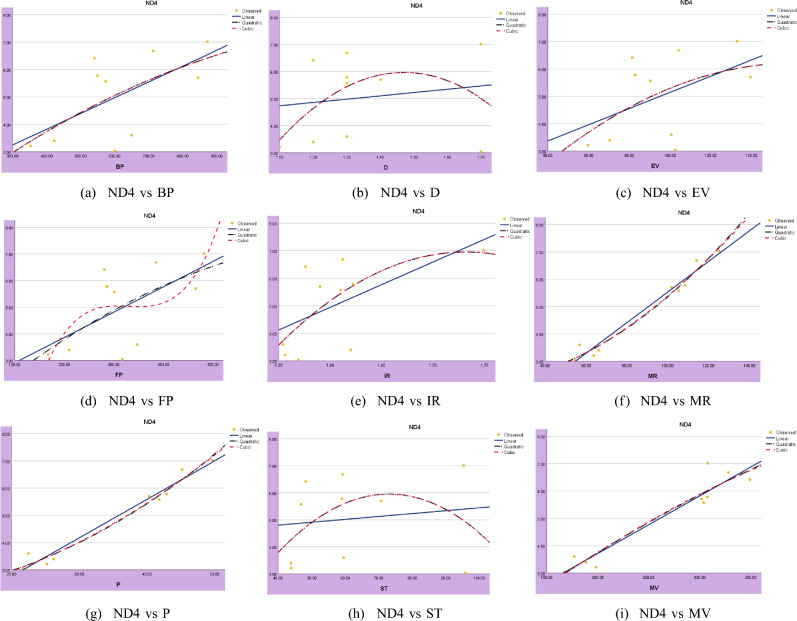
Regression plots corresponding to ND4 index.

**Figure 9:**
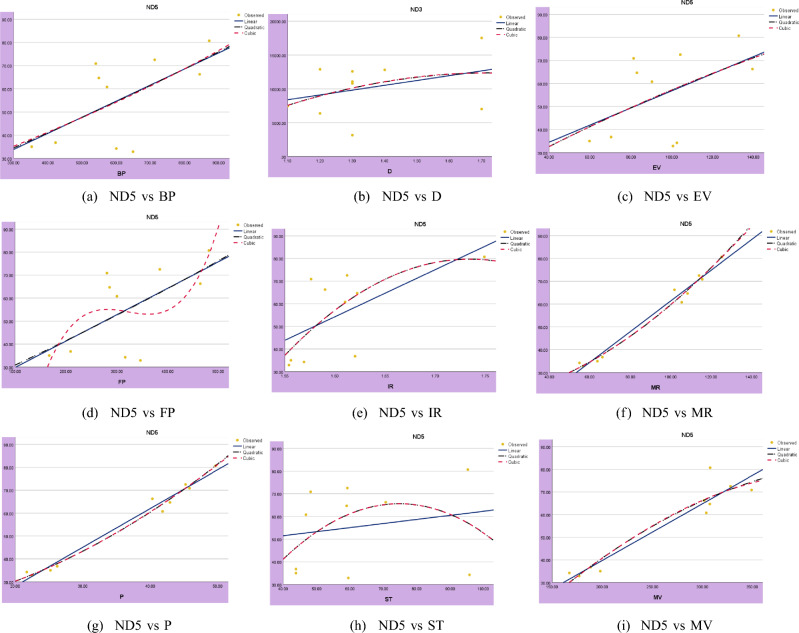
Regression plots corresponding to ND5 index.

Graphical representations of the regression analyses for different indices using logarithmic, quadratic, and cubic models. Figure 3 shows the regression plots of the neighborhood second Zagreb index ($NM_2$), which represents how every model explains the relationship between the neighborhood second Zagreb index and the independent variable. Figure 4 shows the regression analysis that relates to the forgotten topological index (FN1), where the regression shown in the graph indicates the accuracy of each model and the relationship between them. The first NDe index regression model is shown in Fig. 5. Figure 6 also shows graphs that represent the relationship between the second NDe index and the dependent variable. Figure 7 represents the regression analysis of the third NDe index and variables. Figure 8 shows the accuracy with which each model describes the connection between the fourth NDe independent variable and the dependent variable. Figure 9 shows the regression analysis for the fifth NDe index, which shows how well each model captures the connection to the dependent variable.

## Cross validation methodology

Due to the small dataset size (*n = 10*), Leave-One-Out Cross Validation (LOOCV) is applied to evaluate the robustness of the regression models. In LOOCV, one observation is used as a test set while the remaining observations *n-1 = 9* are used for training. This process is repeated for all observations. Model Robustness was evaluated by using cross-validation coefficient determination $(Q^{2})$. if the value of $Q^{2}$ is greater than 0.5, then it will be acceptable for internal predictively. The LOOCV statistics for each physicochemical endpoint are displayed in Table 7. We have performed the whole process using python code. Three properties are predicted such as Molar refractivity (MR), Polarizability (P), and Molar volume (MV). first, Molar refractivity (MR) was observed from SPSS and we obtained the value of $R^{2}$ was 0.980and when we apply cross-validation, then we got the value of $R^{2}$ is 0.9799 and the value of $Q^{2}$ is 0.9691 . Similarly, you can observe the all values which we got from SPSS and Python code are close to each other. This indicates our predicted models are verified by cross validationin table 7.

**Table 7:**
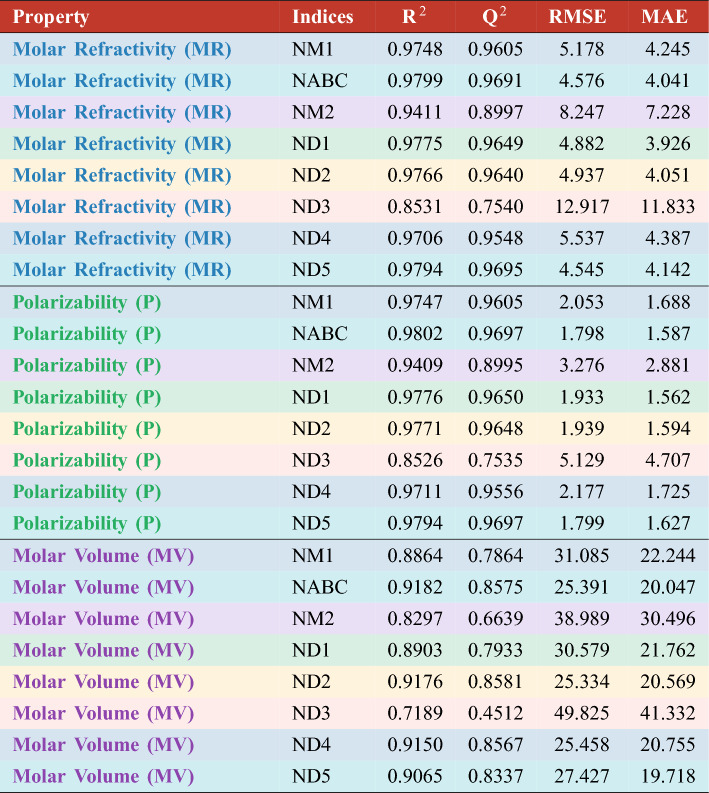
Leave-one-out cross-validation results for optimal predictive indices.

It is to note that the LOOCV procedure was applied to al BP, D, EV, FP, IR, and ST yielded negative or very low $Q^2$ values and were excluded from the LOOCV summary the three physicochemical endpoints: Molar Refractivity (MR), Polarizability (P), and Molar Volume (MV) exhaustively to all the topological indices of the candidate molecules. In fact, a few more properties were also measured, but these returned negative $Q^{2}$ values and so were not pursued further. Many of the indices had negative or very low $Q^{2}$ values, which are not acceptable for the predictive value. To ensure the models used were very robust and very predictive on the internal market, a more stringent criterion ( $Q^{2} > 0.7$ ) was used in this study. For this reason, all indices that did not meet this criterion were explicitly omitted from the table so as to ensure maximum statistical rigor and avoid the misrepresentation of statistics that might not be reliable. Hence, only the most reliable structure–property correlations are reported in Table 7 and this is done for the optimal topological indices that managed to pass the criterion $Q^{2} > 0.7$ for LOOCV statistics.

## Conclusion

This research shows that the topological indices of neighborhood degree are useful instruments in the QSPR framework in predicting physicochemical properties of drug molecules. These findings point out that NABC and ND2 are the most accurate predictors, where it has been found that high accuracy is exhibited in terms of molar refractivity and polarizability, and better performance of the nonlinear models. The research is however confined by the limited data and no large scale validation by which the model generalizability and reliability may be influenced. All in all, the results verify the possibilities of these descriptors to predict the molecular properties efficiently and offer a foundation of future research in the computational drug design. Future research can expand on this contribution by experimenting with the same neighborhood-based indices on a more extensive panel of drug molecules with a more diverse structure. Other regression techniques, including support vector machines or random forests, could also be used by researchers to determine whether the accuracy of prediction is better. The other helpful move would be to come up with the new neighborhood related descriptors that would be able to reflect even more specifics of the molecular shape and bonding.

## Data Availability

The datasets used and/or analysed during the current study available from the corresponding author on reasonable request.

## References

[1] Ravi, V. & Chidambaram, N. QSPR analysis of physico-chemical and pharmacological properties of medications for Parkinson’s treatment utilizing neighborhood degree-based topological descriptors. *Sci. Rep.* **15**, 16941 (2025). 40374678 10.1038/s41598-025-00898-3 PMC12081861

[2] Abubakar, M. S., Aremu, K. O., Aphane, M. & Amusa, L. B. A QSPR analysis of physical properties of antituberculosis drugs using neighbourhood degree-based topological indices and support vector regression. *Heliyon* (2024). 10.1016/j.heliyon.2024.e28260 PMC10987931 38571658

[3] Balasubramaniyan, D., Chidambaram, N., & Ravi, V. QSPR Analysis of Anti-asthma Drugs Using Some Recent Neighbourhood Degree-Based Topological Descriptors. In International Conference on Nonlinear Dynamics and Applications (pp. 380-397). Cham: Springer Nature Switzerland. (2024)

[4] Zaman, S., Majeed, M. U., Ahmed, W. & Saleem, M. T. On neighborhood eccentricity-based topological indices with QSPR analysis of PAHs drugs. *Measure. Interdisciplinary Res. Perspectives* **23**(3), 199–212 (2025).

[5] Altassan, A., Saleh, A., Alashwali, H., Hamed, M. & Muthana, N. Exploring QSPR in breast cancer drugs via entire neighborhood indices and regression models. *Sci. Rep.* **15**(1), 26683 (2025). 40696006 10.1038/s41598-025-12179-0 PMC12284210

[6] Raza, A., Rasheed, M. W., Mahboob, A. & Ismaeel, M. Neighborhood face index: a new quantitative structure property relationship (QSPR) approach for predicting physical properties of polycyclic chemical compounds. *Int. J. Quantum Chem.* **124**(24), e27524 (2024). 10.1038/s41598-024-54962-5 PMC10963771 38528019

[7] Kara, Y., Özkan, Y. S., Ullah, A., Hamed, Y. S. & Belay, M. B. QSPR modeling of some COVID-19 drugs using neighborhood eccentricity-based topological indices: A comparative analysis. *PLoS ONE* **20**(5), e0321359 (2025). 40392879 10.1371/journal.pone.0321359 PMC12091765

[8] Das, S., Rai, S. & Kumar, V. On topological indices of Molnupiravir and its QSPR modelling with some other antiviral drugs to treat COVID-19 patients. *J. Math. Chem.* **62**(10), 2581–2624 (2024).

[9] Tamilarasi, W. & Balamurugan, B. J. QSPR and QSTR analysis to explore pharmacokinetic and toxicity properties of antifungal drugs through topological descriptors. *Sci. Rep.* **15**(1), 18020 (2025). 40410226 10.1038/s41598-025-01522-0 PMC12102203

[10] Kirmani, S. A. K. & Ali, P. Topological descriptors-based QSPR analysis of quercetin derivatives and their application in dextran-quercetin conjugates targeted against cancer treatment. *Chemical Methodologies* **8**(12), 904–929 (2024).

[11] Arockiaraj, M., Godlin, J. J. & Radha, S. Comparative study of degree and neighborhood degree sum-based topological indices for predicting physicochemical properties of skin cancer drug structures. *Mod. Phys. Lett. B* **39**(25), 2550106 (2025).

[12] Akrom, M., Sutojo, T., Pertiwi, A., Rustad, S., & Dipojono, H. K. Investigation of best QSPR-based machine learning model to predict corrosion inhibition performance of pyridine-quinoline compounds. In Journal of Physics: Conference Series (Vol. 2673, No. 1, pp. 012014). IOP Publishing (2023).

[13] Rahul, M. P. & Clement, J. QSPR analysis of carbon allotropes by employing molecular descriptors and information entropies. *Ain Shams Eng. J.* **14**(11), 102542 (2023).

[14] Augustine, T. & Santiago, R. On neighborhood degree-based topological analysis over melamine-based TriCF structure. *Symmetry* **15**(3), 635 (2023).

[15] Jyothish, K., Roy, S. & Gayathri, K. B. Comparative analysis of prediction models for the energetic properties of the trigonal triphenylenoid family using neighborhood degree-based topological descriptors and entropy measures. *Chem. Pap.* 1–13 (2025).

[16] Ayers, M. ChemSpider: the free chemical database (2012).

[17] Hagberg, A., Swart, P. J. & Schult, D. A. Exploring network structure, dynamics, and function using NetworkX (Los Alamos National Laboratory (LANL, 2007) (No. LA-UR-08-05495; LA-UR-08-5495).

[18] IBMCorp Ibm, S. P. S. S. statistics for windows, version 25.0. Armonk, NY: IBM Corp. (2017).

[19] Khalid, W. & Yousaf, S. Neighborhood first Zagreb index and maximal unicyclic and bicyclic graphs. Communications in Combinatorics and Optimization (2024).

[20] Kulli, V. R. Some new status neighborhood indices of graphs. International Journal of Mathematics Trends and Technology-IJMTT (2020).

[21] Barik, S. & Verma, P. On spectral properties of neighbourhood second Zagreb matrix of graph. *Commun. Combinatorics Optimiz.* **10**(2), 275–293 (2025).

[22] Ali, H., Babar, U., Arshad, S. H. & Sajjad, A. On some neighbourhood degree-based indices of graphs derived from honeycomb structure. *Konuralp J. Math.* **9**(1), 164–175 (2020).

[23] Mondal, S., De, N. & Pal, A. On some general neighborhood degree based topological indices. *Int. J. Appl. Math.* **32**(6), 1037 (2019).

